# The Past Ubiquity and Environment of the Lost Earth Buildings of Scotland

**DOI:** 10.1007/s10745-017-9931-4

**Published:** 2017-09-15

**Authors:** Simon J. Parkin, W. Paul Adderley

**Affiliations:** Biological and Environmental Sciences, Faculty of Natural Sciences, University of Stirling, Stirling, Scotland FK9 4LA UK

**Keywords:** Earth-building, Turf, Mudwall, Vernacular architecture, Climate change, Scotland, British Isles

## Abstract

This paper investigates the once ubiquitous vernacular earth-built structures of Scotland and how perceptions of such buildings were shaped and developed through periods of intense cultural and environmental change. We focus upon the past exploitation of traditional resources to construct vernacular architectures and on changes in the perception of the resultant buildings. Historic earth-built structures are today deeply hidden within the landscapes of Scotland, although they were once a common feature of both urban and rural settlements. Whilst the eighteenth and nineteenth century period of Improvement – during which many of these structures were destroyed, repurposed, or left to decay – has received extensive attention by historians, there exists no previous serious study of the human and environmental dimensions. Through analysis of the material aspects of landscape resource use and analysis of the historical perceptions of such use, we emphasize the national significance of this undervalued aspect of Scotland’s built and cultural heritage, increasingly at risk of being lost completely, highlighting the prior ubiquity of mudwall structures.

## Introduction

Earthen materials - soil, unfired clay, and turf - have been used in a variety of ways and in conjunction with numerous other materials for millennia in the construction of vernacular buildings throughout Europe. In the British Isles, even when not used as the primary structural element, surveys reveal clay-rich subsoil employed to reduce friction between massive stone slabs in prehistoric tombs, applied to wallheads as a form of waterproofing, or as mortar in stone-built structures from farmhouses to castles (Walker *et al.* 1996). Today, these materials and techniques are increasingly being re-examined by engineers and architects as part of an ecologically-aware building agenda (Guillaud and Houben 2006).

There are commonalities in many earth building techniques across Europe. Our consideration of earth-built vernacular architectures in Scotland, therefore, has many parallels (See for example Klapste 2002). However, the great variation in Scotland’s geology and surface soils is reflected in the variety of ways earth material has been employed as a constructional component, with notable subtleties within smaller localities (McGregor 2010). Examining a range of Scottish contexts therefore allows consideration of changes in a variety of different construction traditions during a common period of past social change.

**Fig. 1:**
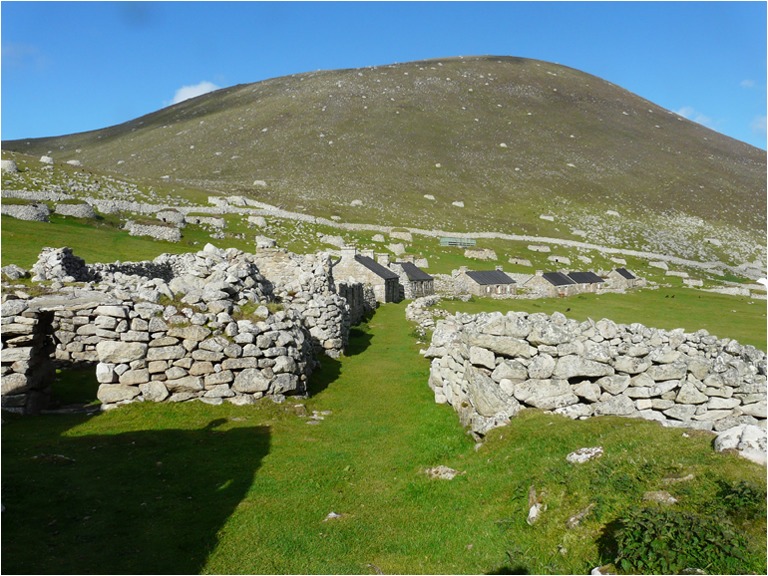
Stone walled buildings constructed ca. 1860, St Kilda to replace previous earth-built structures

Turf and, to a lesser extent, peat, represent a major material component in this building tradition (Walker *et al.* 2006). Whether employed alone as a walling material or in combination with alternating stone layers (Fenton 1968), as roofing or applied to wattle-work (Walker *et al.* 2006), turf was an omnipresent and, given a long enough period, renewable resource. The blackhouses synonymous with the Western Isles of Scotland, which, with their walls of turf or a double skin of dry stone filled with loose earth, were developed in response to a harsh North Atlantic environment. Representing a different tradition to that of the Norse longhouse (Walker *et al.* 2006), Geddes (2013) even suggests that these blackhouses form the later part of a practice that encompasses Iron Age broch structures. The human ecology expressed here, where vernacular construction traditions are seen as a response to specific environmental conditions, is further emphasized by the relative inadequacy of the replacement dwellings imposed from the 1860s on the remote North Atlantic island community of St Kilda (Fig. 1; Carruthers and Frew 2003).

Across Scotland by the late-eighteenth century turf structures were seen as the lowest form of available shelter, with the influential Improver Sir John Sinclair of Ulbster calling for their construction to be prohibited. His reasons were threefold, noting the loss of potential agricultural land (a late-twentieth century turf-house reconstruction at the Highland Folk Museum used an acre of turf (Noble 1983)); the impermanence of the wall material; and the damp and “unwholesome” living conditions that such homes created (Sinclair 1795). Common in eighteenth century travelers’ diaries are references to turf dwellings as ‘hovels,’ for instance Edmund Burt when describing parts of Inverness (Simmons 1998) and Thomas Pennant when commenting on rural Sutherland (Pennant 1776). Such interpretations influenced the perception of vernacular earth buildings more generally, suggesting low quality, impermanent structures.

**Fig. 2:**
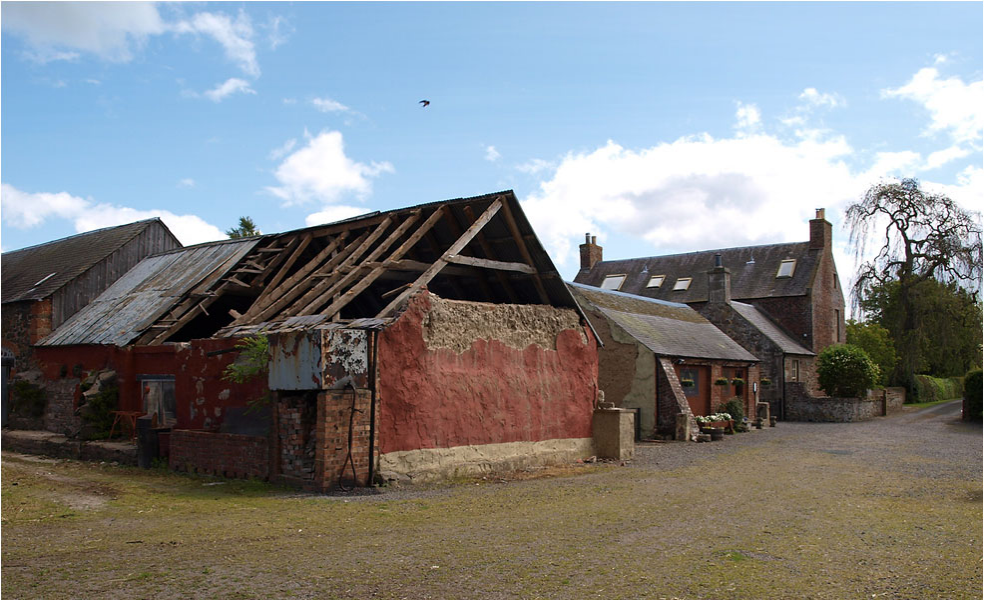
Flatfield Steading, Perthshire. Later-eighteenth century group of farm buildings, two of which are of mudwall. These were cement-rendered in the 1970s to the detriment of the walling fabric

**Fig. 3:**
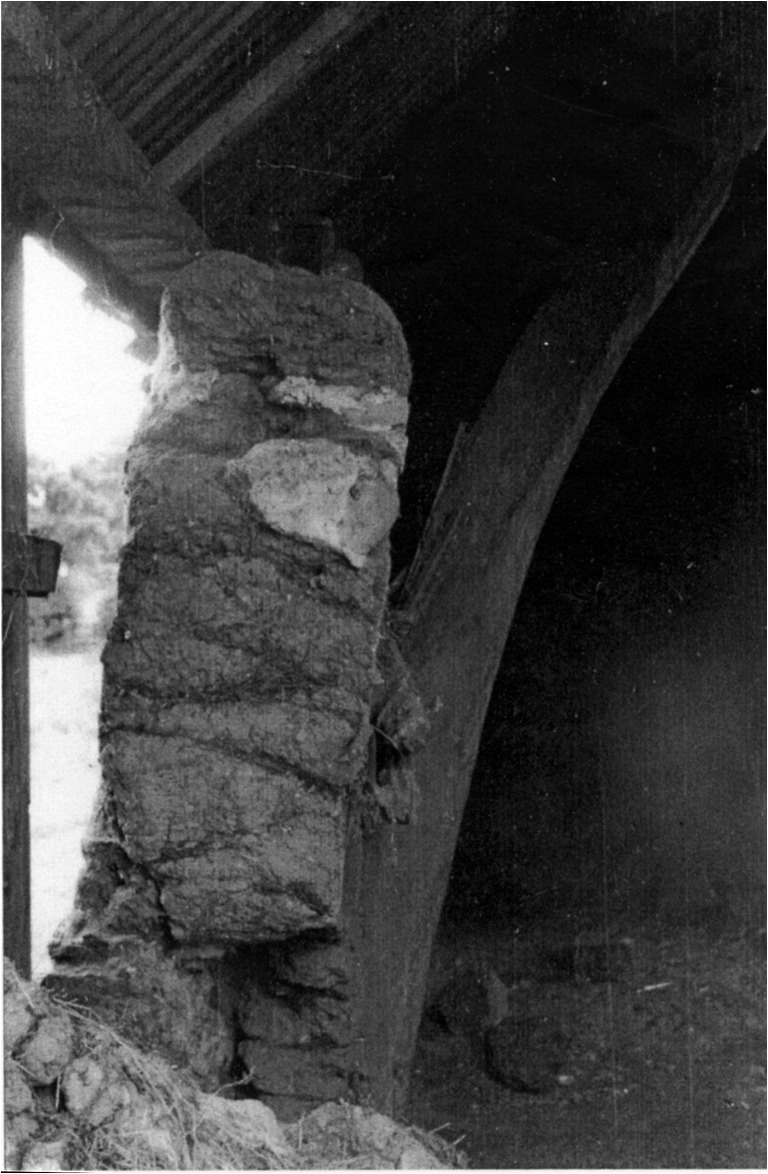
Mudwall with straw layers included between lifts. Prior Linn farm, Canonbie, Dumfries and Galloway (taken by Werner Kissling in 1954 and held by the School of Scottish Studies Archive, University of Edinburgh)

**Fig. 4:**
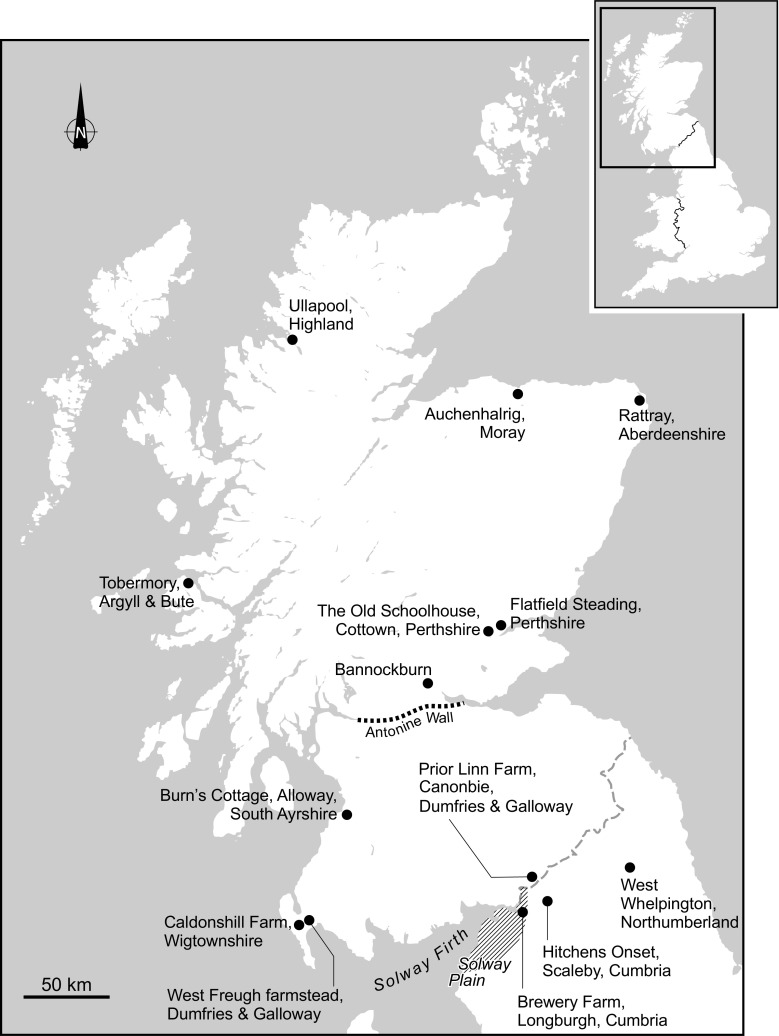
Map of Scotland and the north of England showing site locations discussed

This criticism is not restricted to turf as a construction material and our primary focus here is on the mass earth type of building tradition that was common throughout Scotland. This method saw a mineral subsoil, typically mixed with fibrous matter such as straw, hair, or heather, gravel (though not always) and water, built in layers (or lifts) on top of a low rubble or stone base until a monolithic wall of requisite height resulted (Fig. 2). The vagaries of Scotland’s geology and resultant soils, economy, and culture produced a variety of outcomes in practice such that there is no definitive ratio of materials used across contexts (Stell 1993). Auchenhalrig Work, also known as ‘Clay and Bool’ or ‘Straw and Dash,’ a tradition distinct to the locality around a village in Morayshire in Northern Scotland from which the name derives, used mass earth in combination with prominent rounded boulders. In some instances, straw would be laid between lifts (Fig. 3) with dung, blood, urine, or other organic additives perhaps used to modify cohesion and workability. Surviving evidence of mass earth construction can, therefore, be found across many Scottish landscapes (Fig. 4). Mass earth construction is widely known as ‘cob,’ a term recognized in the south-west of England and in many generalized texts, but also referred to under a variety of different regional terms including ‘clom’ in Wales, ‘dabbins’ in Cumbria, England, and ‘mudwall’ in Scotland.

The reasons why such a drastic decline in the use of both turf and mudwall took place in Scotland over a period covering three centuries from the mid-1700s have typically been seen as part of the narrative of Improvement, which suggests the imposition of an architecturally-designed building stock from pattern books as a corollary to agricultural development (Walker 1979). Agricultural revolution is seen to be followed by industrial advancement, the emergence of a more all-encompassing economy and increasing availability of cheaper imported building materials following the advent of extensive railway networks (Table 1). This meant that local context, climatic conditions, and the lifestyles of the rural population were no longer the underlying rationale for building methods or, indeed, materials utilized (Carruthers and Frew 2003). Scotland’s landscape today is a product of these developments with stone-built buildings and crofting settlements typically viewed as romantic remnants of past times. The reality, however, is that the many stone farmhouses, with their homogenized appearances across the regions of Scotland, are generally part of a relatively modern eighteenth and nineteenth century aesthetic that severed links to traditional vernacular buildings and the methods of their erection (Fenton 2008; Richards and Richards 1994). Similarly, the crofts created by the displaced communities of the Improvement are also of this period. Today, these crofts are seen to be representative of traditional life and vernacular construction despite their relatively recent introduction.

**Table 1:** Timeline relating key events with chnages in urbanization and land manangement of Scotland through the periods of Improvement and industrialization

Decade	Events	Urbanization	Land
1671			
1681			
1691			
1701	1707 Act of Union (English and Scottish Parliaments combine)		
1711	1715 Jacobite Rebellion		
1721			
1731			1739 and 1740 Grain harvest failures
1741	1745/6 Jacobite Rebellion		
1751			
1761	1765 First developments of Edinburgh’s New Town	Industry-led urban expansion	Wide-spread investment in Improvement; Land enclosure; Increased adoption of single-farm tenancies
1771	1776 Adam Smith’s “The Wealth of Nations” published		1776 Kames’ “The Gentleman Farmer” published
1781			
1791	Napoleonic wars stimulate domestic agricultural and industrial production		1793 Board of Agriculture established
1801		17% Population in urban contexts of 10,000 people or more	Period of Highland Clearances (c.1800 – c.1850)
1811			1811 Peak of agricultural employment
1821			1828 - Scottish wool production <10% of UK Total
1831			
1841	First major railways established facilitating increased transport of building stone	>30% Population in urban contexts of 5000 people or more)	c. 1840 - Scottish wool production >25% of UK total
1851		43% of working population employed in manufacturing	
1861			
1871			
1881			
1891			
1901			
1911	The Great War	>60% Population in urban contexts of 5000 people or more	

The instincts of many present-day heritage organizations appear to remain focused on evocative buildings. Consequently, such organizations may overlook ‘lowly’ constructions unless in dramatic or romanticized settings. However, it is in vernacular buildings within mainland rural settings that the experiences of the vast majority of Scotland’s past population are found. Perceptions of materials used also change; stone, which was deemed a necessary means of improving the building stock by the later eighteenth century, was often used earlier as a vernacular material. Fenton (1997) has postulated that Orkney probably retains a greater proportion of ancient farm buildings than elsewhere in Scotland due to the flagstone beds of Old Red Sandstone that provided durable structures distinct from those often built contemporaneously with perishable earth walls across much of the mainland.

Consideration of Scotland’s vernacular heritage has been the reserve of a small body of scholars, with an emphasis on surveying surviving examples (Walker 1977, 2006). Our approach here is therefore to incorporate evidences from the rest of Britain, especially where a cultural continuity of practice is evident. Fenton and Walker’s (1981) recording of the variety of vernacular constructions found across Scotland was vital to cataloguing an ever-decreasing array of structures. There remain, however, significant gaps in our understandings of these structures in a wider historical context. By developing further insights into these constructions, the environment in which earth buildings were found in rural Scotland can be considered and the changing perceptions of earth buildings revealed.

## Earth-Building Materials, Construction, and Maintenance

**Fig. 5:**
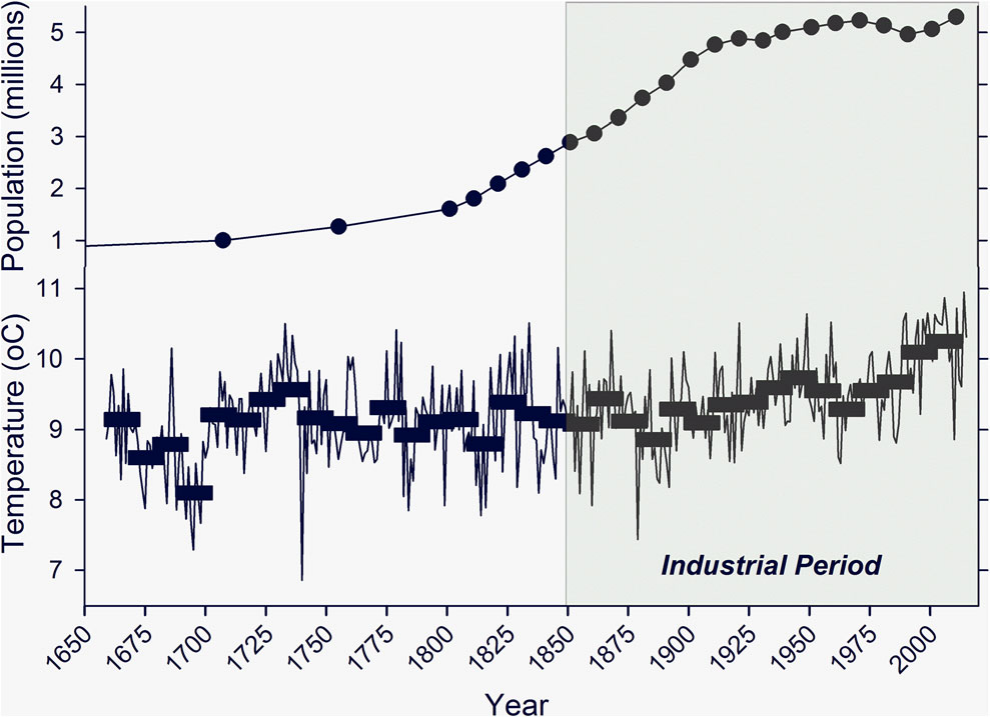
Central England climate record with decadal mean temperature values plotted against mean annual temperatures.  Population of Scotland plotted from census data and earlier estimates.   Data are abstracted from Parker et al. 1992; and from UK Met Office Hadley Centre (http://www.metoffice.gov.uk/)

Fenton (2008) associated pre-Improvement building practices with pure functionality and outlined a key point that local earth-based materials were taken from the environment and utilized before degrading and returning from whence they came, thus making them an inherent part of the systems of traditional rural life in Scotland and northern England. The rapid climate changes reported for the late twentieth and early twenty-first centuries are undoubtedly an influence on the types and rates of processes involved in the degradation of earthen building materials. Taking a longer view we can consider the compiled set of monthly temperature records for central England as offering the most comprehensive data set for northern European temperatures of the seventeenth century onwards (Fig. 5). This suggests, for the period prior to late nineteenth century industrialization evidenced by rapid population growth, that the extremes of temperature seen within each decade exceed any decade to decade shift. The cycle outlined by Fenton, therefore, can be considered independent of the effects of long-term temperature change. The notion that building with earth is part of a material cycle and can withstand future climate change is one that pervades in thinking that has brought about its recent revival, although successive generations of Scotland’s inhabitants recognized this as part of their agricultural regimes from prehistory through to the nineteenth century.

**Fig. 6:**
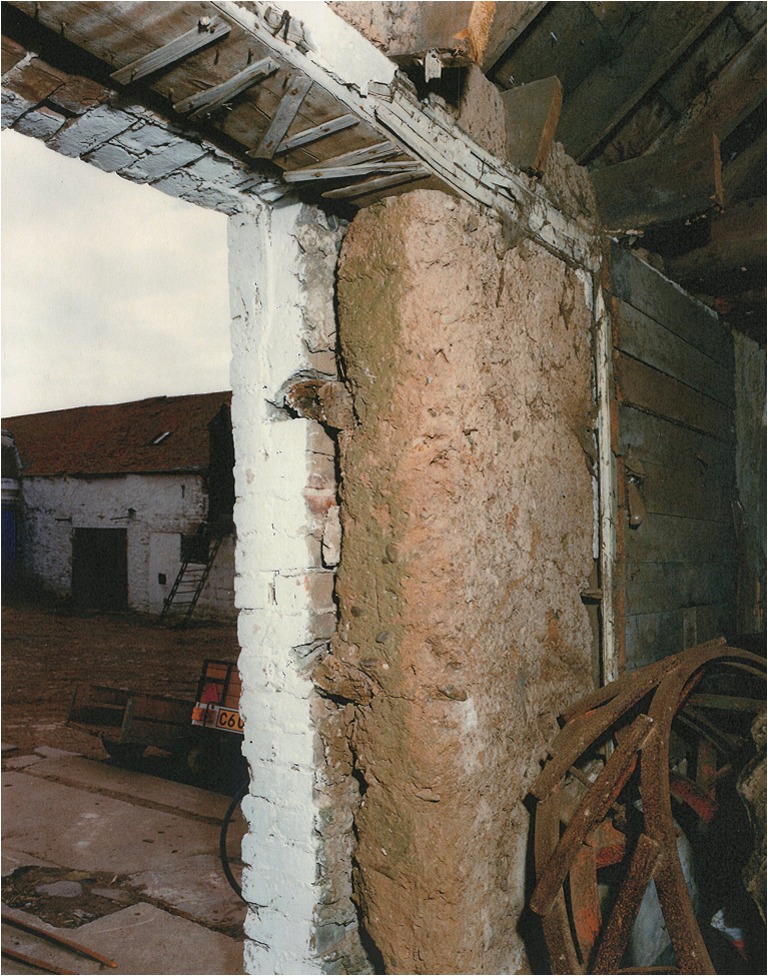
Brick-faced claywall steading, Caldonshill Farm, Dumfries and Galloway, © Crown Copyright: RCAHMS. Licensor www.rcahms.gov.uk

Use of the words ‘clay’ and ‘mud’ occurs in studies of Scotland’s earth building tradition and the contexts in which they were employed can be traced in part through an assessment of the Dictionary of the Scots Language (2004) (Table 2). The words mudwall and claywall are most commonly used to describe mass earth structures in Scotland. McGregor (2010) has emphasized the significance of place when associating mudwall with Dumfriesshire and north-west England around the Solway Plain whilst Maxwell (1996) chooses to regard the material of mass earth walled structures of the north-east, the Carse of Gowrie, and the Solway area as clay, without reference to mud. Also in modern field surveys, when a mass-earth wall is found faced (though not bonded) with another material, such as brick, it is known as ‘claywall’ (Walker *et al.* 1996). Thus, when examining the Scottish landscape for mass-earth walled buildings it becomes apparent that a greater number of examples may still exist, since buildings may be faced in a second, presumably later, skin of more ‘permanent’ material such as stone or brick. The *Royal Commission on the Ancient and Historic Monuments of Scotland* (*RCAHMS*) has surveyed examples of this, such as at Caldonshill Farm in Dumfries and Galloway where a claywall steading faced in later brickwork still stands (Fig. 6). Another example in Leetown, Perthshire, has experienced accelerated degradation in recent years.

**Table 2:** Terms used in the description of Scottish vernacular earthen architectures and building materials

Modern Description	Terminology from the Dictionary of the Older Scottish Tongue (twelfth to seventeenth centuries)	Terminology from the Scottish National Dictionary (eighteenth to twentieth centuries)
Mudwall (Cob)	“Mude-, Mudwall... A wall built of mud or clay; the material forming such a wall”.	
Clay	“Clay... To smear or plaster with clay”	
Straw	“Cat... A wisp of straw combined with soft clay used in building or repairing walls”	“*clay-cat*, *−kat*, a bunch of straw mixed with clay used in the building of a mud wall”
Specialist in mudwall earth construction		“*clay-an’-dubber*, a builder of houses with mud walls; one who does *cat-an’-clay* work
Clay-bonded thatch roofing		*clay-thack*, thatch held in position by clay”

Usage of the words clay and mud as raw materials was transposable (National Archives of Scotland (NAS) GD139/110), meaning that the sources may equally refer to clay mortar and mud mortar, clay walls and mud walls. The compound ‘claywall’ is not prevalent in archival material, whereas ‘mudwall’ is; this distinction suggests that the former is a more recent introduction

It is important to emphasize that historic earth-built vernacular structures could be made entirely permanent when appropriately maintained with regular new thatch and lime harl. Therefore, the propagation of negative perceptions of vernacular mudwall dwellings must be partly explained by disparities in the quality of build and suitability of external finish. It may thus be assumed that many mudwall buildings of ‘lower’ status were left unrendered upon completion, leaving them unprotected from water infiltration and wind erosion and so liable to be lost. A further qualification is that the relative ratios of cost and maintenance, rather than longevity of a building’s life-cycle, can determine perceptions of permanence. Thus, the notion follows that true permanence is determined by high initial and low maintenance costs, whilst semi-permanence is defined through low building cost with a need for frequent repair (Wrathmell 1984). This defines the essential nature of historic earth-built structures that a disconnection to such regimes within modern Western society likely influences the perception of vernacular tradition.

## Earth Buildings in the Pre-Improvement Scottish Landscape

Although there can be little doubt as to the ubiquity of earth-built structures in Scotland prior to the eighteenth and nineteenth centuries, proving this earlier prevalence through survey, archival, or archaeological record poses difficulties. Indeed, it has been noted that archaeological remnants of rural dwellings from the Iron Age are more prominent in the Scottish landscape than those of the sixteenth century. With extant lay buildings pre-dating the mid-eighteenth century virtually unknown, a common assumption is that poor construction and the nature of materials used resulted in buildings with short lives (Whyte and Whyte 1991). Dyer (1986), as part of the revision of Hoskins’ theory of the ‘Great Rebuilding', has speculated on the impact that building with crucks (‘couples’ in Scotland) may have had on the quality of peasant buildings, emphasizing the importance of the introduction of stone foundations. Employed as a means of protecting timbers from rotting, these low walls were also ideal for mudwall construction, limiting the capillary rise of groundwater that is otherwise deleterious to earthen building materials. Although the assumption may be that the majority of medieval peasant buildings’ walls were infilled with wattle and daub, there seems no great hurdle to overcome in considering the use of mudwall at an earlier time than sources can tell us for regions in which it is known to have become common. Longcroft (2006) has noted the growing body of archaeological evidence for buildings with massed earth walls from the thirteenth and fourteenth centuries from southern England to Wales, Northern Ireland, and Scotland, whilst testifying to the presence of such buildings in both rural and urban Norfolk from at least the eleventh century. Research into the history and nature of earth building around the Solway Plain, in north-west England, has been conducted by Brunskill (1962), Harrison (1989, 1991), and Jennings (2002), all giving recognition to the continuity of this tradition into south-west Scotland. More recently, a survey of 312 sites with complete or partial remains of historic clay dabbins in Cumbria has resulted in a reappraisal of the origins of this building tradition. Previously dated to no earlier than the seventeenth century, there is evidence amongst the surviving 306 buildings that these small dwellings and agricultural buildings were constructed from at least as early as the fifteenth century. Examples have been identified at Brewery Farm, Longburgh and Hitchens Onset, Scaleby, whilst another at Ratten Row, Durdar, dates from around 1505 (Oxford Archaeology North 2006).

**Fig. 7:**
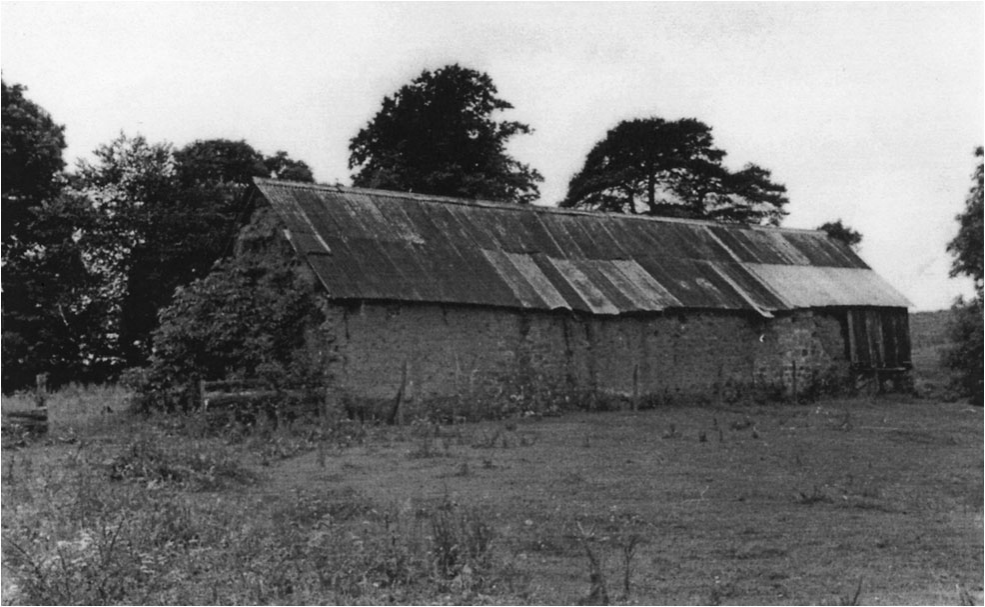
External view of mudwall steading at Prior Linn Farm, Canonbie, Dumfries and Galloway (taken by Werner Kissling in 1954 and held by the School of Scottish Studies Archive, University of Edinburgh)

Elucidating the impact of the emergence of cruck building in England, Wrathmell also alluded to the tendency of scholars to consider medieval peasant building as impermanent and of poor quality, therefore explaining the lack of surviving examples prior to the introduction of ‘improved’ farmhouses in the sixteenth and seventeenth centuries. Taking an archaeological perspective, Wrathmell (2002) reasons that following abandonment of settlement sites vernacular walling materials would have inevitably disappeared and structural timbers been reused elsewhere as a matter of economic sense. This assertion particularly reflects the increasing dearth of good quality timber. In Scotland, the shortage of timber was causing concern to Parliament in the sixteenth and seventeenth centuries and led to a series of Acts for the protection of the resource (Smout et al. 2005). Such factors surely put greater emphasis on the need to build decent quality buildings with alternative, cheap, durable materials, of which well-built mudwalls would be one. Dixon (2002) has discussed the emergence of cruck building in lowland Scotland in the thirteenth to fourteenth centuries, whilst archaeological evidence from the deserted medieval settlement of Rattray in Aberdeenshire revealed fourteenth century clay- and clay and rubble-walled buildings (Murray and Murray 1993). These structures appeared to succeed thirteenth century buildings that were primarily of wood and the authors of the excavation report postulate that this may have been directly correlated with the exhaustion of local timber supplies. Standing survivals of cruck-framed mudwall buildings can be found in Scotland, such as at Prior Linn Farm (Figs 2 and 7), which can be viewed as part of the wider building tradition around the Solway Firth.

## Perceptions Prior to Improvement

Perceptions of Scottish vernacular buildings have undoubtedly developed and altered over time, although the attitudes of those who historically built and lived in vernacular contexts are rarely known. In the late-nineteenth century, antiquarian Peter Hume Brown compiled a series of accounts made by foreign visitors to Scotland prior to the eighteenth century, including that of Thomas Morer. Brown’s collection was based on printed sources rather than original manuscripts and it can be assumed that a number of the visitors’ generalized descriptions were based on limited stays in the country or the plagiarism of other past descriptions. However, these can still be of value as early travelers’ accounts of Scotland (Rackwitz 2007).

The earliest account in the collection is from Æneas Sylvius Piccolomini, the future Pope Pius II, who in the mid-fifteenth century painted what became a fairly typical outsiders’ view of Scottish lay dwellings. His description notes that “the roofs of the houses in the country are made of turf, and the doors of the humbler dwellings are made of the hide of oxen” (Hume Brown 1891). Although overt judgments of these vernacular building materials are avoided, use of the term ‘barbaros’ clearly emphasized links between Scottish society and living conditions with backwardness and a lack of civilization (Rackwitz 2007).

Thomas Kirke added to earlier accounts by specifying some of the materials typically employed in common dwellings. *A Modern Account of Scotland by an English Gentleman*, published in 1679, is unsympathetic in describing the living conditions of the bulk of Scotland’s population: “The houses of the commonalty are very mean, mud-wall and thatch the best; but the poorer sort live in such miserable huts as never eye beheld; men, women and children pig altogether in a poor mouse-hole of mud, heath and some such like matter; in some parts, where turf is plentiful, they build little cabins thereof, with arched roofs of turf, without a stick of timber in it; when their houses are dry enough to burn, it serves as fuel, and they remove to another” (Hume Brown 1891). Kirke asserts the ubiquity of mudwall and turf building in Scotland by relating them to the houses of the masses and, although he inevitably regards all of their dwellings as “very mean,” his opinion that mudwall homes were superior to the other types of earth-built structures seems creditable. Kirke’s descriptions are filled with diatribe, concealing the reality of his experiences, which he recorded in diary entries during a three-month tour in 1677 that were straightforward and free of the negative embellishment found in the published work. As Rackwitz noted, the diary reflects well on Scotland generally, being both appreciative and praising, meaning that the version presented in his *Account* was created for the benefit of an audience familiar with anti-Scottish works such as those of Sir Anthony Weldon (Rackwitz 2007).

## Prior to Improvement – The Bannockburn Papers

**Fig. 8:**
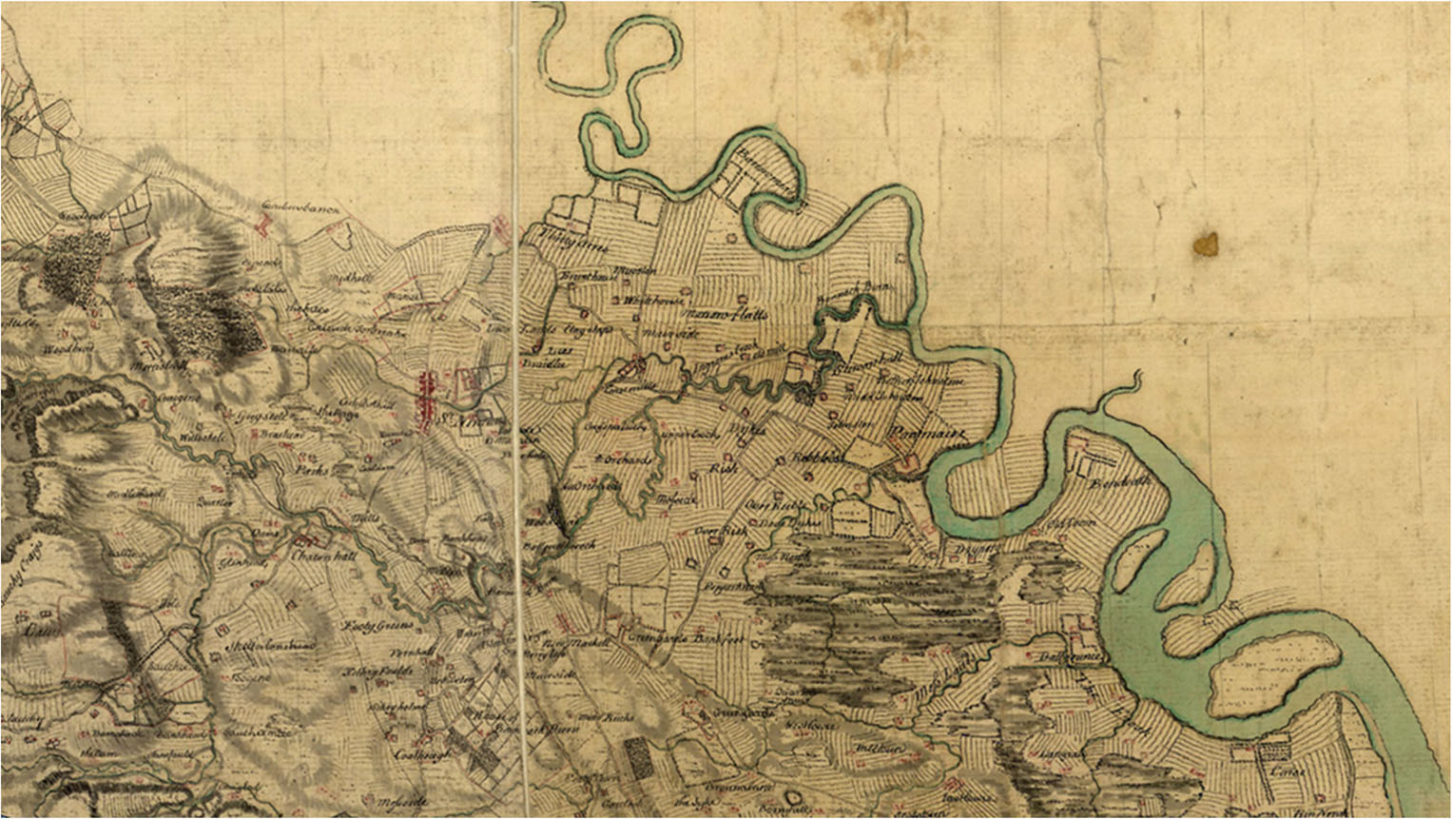
The settlement at Bannockburn is shown by Roy in the 1750s as being within agricultural land (spanning break in map sheets) with a discrete area of bog peatland lying to the east. (Roy Military Survey of Scotland 1747–1755. © British Library Board, British Library Maps C.9.b 6/7a and C.9.b 6/7b.)

A new insight into the extent of mudwall dwellings and agricultural structures in Scotland is afforded by the post-1715 Forfeited Estate Papers for Bannockburn, Stirlingshire. The landscape around Bannockburn, part of the Carse of Stirling, is extremely low-lying and, like the nearby Kincardine Moss, renowned for having been cleared of its extensive cover of peat over a number of decades from the 1760s. The Carse of Stirling was not composed of peat-topped bogs alone, however, as many accounts would lead one to believe (Harrison 2003), and a patchwork of agricultural and moss lands stretching from the Upper Forth were attested to in the maps of William Roy in the mid-eighteenth century (Fig. 8), crucially, prior to the advent of drainage and clearance. The Carse of Stirling has widespread clay soils (buried where great depths of peat were and still are found) and Stirling gives its name to the clay soil Association found locally and that covers over 400 km2 of Scotland’s landmass (Wilson *et al.* 1984).

The “Accompt of the Quantitie and Qualitie of the lands tenents and hereditaments Which Belonged to Sir Hugh Patersone” (NAS E616/1 n.d.), dated 19 November 1716, provides details of the lands and buildings held by 60 named tenants and the rentals they paid to the forfeited landowner prior to his death. The list is noticeably split between those named as “feuers” who had holdings “in few” and those who were said to “posess” certain buildings and lands. Of the nine feuers and 51 possessors named a number held multiple dwellings, suggesting that sub-letting was prevalent.

Dyer (1986) suggests that the dimensions of a single bay were typically around 15 ft. by 15 ft. (c. 21 m^2^). Examination of the nature and number of the buildings recorded in the account (Table 3), while assuming each bay as the minimum possible dwelling space, suggests that 38 potential dwellings were built with stone and mud, ranging from a single room to a two-storey house, along with a total of up to 77 mudwall dwellings and 17 mudwall ancillary buildings or extensions. One of the dwelling houses was specified as being “two Couple length,” suggesting a similar building template to medieval structures built with crucks. The descriptions of buildings with multiple bays may also imply the use of cruck construction if it is given that each bay equated to the space between two sets of crucks.

**Table 3:** Analysis of the building types associated with the sets of “Fewars”, “Tenents” and “Posessors” named within the Bannockburn papers

Name	Status	Houses/ other	Barns and byres	No. bays/ length	Materials	Cott houses	Bays	Materials
John Robin	Fewar	ane house two story high	No	about forty foot long	–	Nine	–	All thatched with straw
James Buchan	Fewar	ane laigh thatched house	No	–	stone and mudd; thatched with straw	–	–	–
Archibald Wordie	Fewar	Nine little houses...one whereof is two story high	No	–	stone and mudd; thatched with straw	–	–	–
David Brown	Fewar	ane house	No	two Couple length	stone and mudd; thatched with straw	–	–	–
John Steill	Fewar	ane room of ane thatched house	No	–	stone and mudd; thatched with straw	–	–	–
William Andersone*	Fewar	two little houses	No	–	stone and mudd; thatched with straw	–	–	–
William Keir*	Fewar	two houses	No	–	stone and mudd; thatched with straw	–	–	–
John Loackart*	Fewar	ane little house	No	–	stone and mudd; thatched with straw	–	–	–
Henry Hill*	Fewar	ane little house	No	–	stone and mudd; thatched with straw	–	–	–
William Gillespie*	Posessor	ane little house	No	–	stone and mudd; thatched with straw	–	–	–
James Burden	Tenant	ane little dwelling house	Yes	about four bey	mudd; thatched with straw	–	–	–
John Lourie*	Posessor	ane little dwelling house	Yes	about four bey	mudd; thatched with straw	–	–	–
Andrew Gray*	Posessor without tack	ane little dwelling house	Yes	about four bey	mudd; thatched with straw	–	–	–
William Smith*	Posessor by tack	ane little dwelling house	Yes	about four bey	mudd; thatched with straw	–	–	–
Thomas Smith	Posessor	houses	Yes	three bey	mudd	–	–	–
William Andersone*	Posessor	houses	Yes	three bey	mudd	–	–	–
John Wright	Posessor	houses	Yes	six or seven bey	mudd	–	–	–
John Frizall(?)	Posessor	–	–	–	–	ane	–	–
Alexander Hall	Posessor	–	–	–	–	ane	–	–
John Hall	Posessor	–	–	–	–	ane	–	–
John Andersone	Posessor	–	–	–	–	ane	–	–
Alexander Mackie	Posessor	ane house	Yes	about four bey	mudd; thatched with straw	–	–	–
John Steven*	Posessor	ane house	Yes	about four bey	mudd; thatched with straw	–	–	–
Stepehn Watson	Posessor	ane house	Yes	about four or five bey	mudd; thatched with straw	–	–	–
John Glen	Posessor	ane house; ane malt kiln	Yes	–	mudd; thatched with straw	some cott houses		mudd; thatched with straw
John Johnston*	Posessor	four bey of houses	Yes	–	mudd; thatched with straw	–	–	–
John Jaffray	Posessor	ane sitt house	Yes	about four bey	mudd; thatched with straw	–	–	–
John Burges	Posessor	houses	–	seven bey	muddwall; thatched with straw	–	–	–
William Dow	Posessor	ane house	Yes	about three bey	muddwall; covered with thatch	–	–	–
Alexander Jaffray*	Posessor	ane house	Yes	about three bey	muddwall; covered with thatch	–	–	–
James Atchesone	Posessor	ane little dwelling house; ane walkmiln	–	–	muddwall; thatched with straw	–	–	–
Andrew McGowan	Posessor	houses	–	four bey	muddwall; thatched	–	–	–
Gabriel Andrew	Posessor	houses	–	four bey	muddwall; thatched	–	–	–
John Andrew	Posessor	houses	–	four bey	muddwall; thatched	–	–	–
Duncan Young	Posessor	–	–	–	–	ane little cotthouse	–	muddwall; thatched with straw
John Russall	Posessor	houses	Yes	three or four bey	muddwall; thatched	–	–	–
John Gillespie	Posessor	houses	–	four bey	muddwall; thatched	–	–	–
David Robertsone	Posessor	houses	–	four bey	muddwall; thatched	–	–	–
Helen Logon	Posessor	ane house	–	–	muddwall and stone; thatched	–	–	–
Mareon McFeal	Posessor	ane house		two bey	muddwall; thatched	–	–	–
John Hall	Posessor	houses	Yes	about six bey	mud and stone; thatched	–	–	–
Isobel Russall*	Posessor	houses; ane maltkiln	Yes	about six bey	mud and stone; thatched	–	–	–
James Miller	Posessor	houses		three bey	muddwall; thatched	–	–	–
Margarat Richards	Posessor	–	–	–	–	ane cotthouse	–	–
Robert Stevensone	Posessor	–	–	–	–		–	–
Thomas Taylor	Posessor	–	–	–	–	ane cotthouse	–	–
John Donaldsone	Posessor	–	–	–	–	ane cotthouse	–	–
Agnes Mitchell	Posessor	–	–	–	–	ane cotthouse	–	–
Alexander Drummond	Posessor	–	–	–	–	ane cotthouse	–	–
David Andersone	Posessor	–	–	–	–	ane cotthouse	–	–
John Rae	Posessor	–	–	–	–	ane cotthouse	–	–
John Tannoch	Posessor	ane house	–	–	–	–	–	–
Graham Walker	Posessor	–	–	–	–	ane cotthouse	–	–
Alexander Johnston	Posessor	–	–	–	–	ane cotthouse	–	–
John Lourie	Posessor	–	–	–	–	ane cotthouse	–	–
Edward Hall	Posessor	–	–	–	–	ane cotthouse	–	–
John Richardsone	Posessor	–	–	–	–	ane cotthouse	–	–
Alexander Cowan	Posessor	–	–	–	–	ane cotthouse	–	–
James Murehead	Posessor	houses	–	–	stone and mudd; thatched	–	–	–
William Andersone	Posessor	–	–	–	–	–	–	–

*those marked with an asterix are described as having possessions of the quantity and quality of the previous entry

The Bannockburn account is particularly useful for studying the historical economy and human ecology of earth building in Scotland as the pre-Improvement structure of landholding was probably retained, with some small parcels of estate land still worked for subsistence purposes rather than being consolidated into large enclosures for capital gain. Furthermore, the middling tenants could retain surplus incomes after paying their master with rent from sub-letters (Devine 2006). If the various buildings held by individual feuers and possessors at Bannockburn are considered in conjunction with the lands they worked, inferences can be made about their relative social standing and how this related to the building materials used. Typically, feuers are associated with buildings of mud and stone construction, whereas possessors seem to have predominantly occupied mudwall buildings. This distinction presumably reflects the contrasting arrangements held between these tenants and the landlord and, furthermore, infers that those in possession were directly responsible for their own construction, while the feuers inherited buildings from the estate. Moreover, the possessors appear more likely to have owned “cotthouses” for the purposes of sub-letting.

Declarations dated three days later than the account, by William Dollar, Lilias Cowan, and James Walker, tenants who held 19 year tacks for extensive holdings, are also included in the Bannockburn papers. Dollar and Cowan together held “the miln of Skeock which is two storey high and about fourty foot long built with stone and lyme and sclaited as also two corn kilns with a ruinous maltbarn which wants the roof all built with stone and mudd and thatched with straw, And suchlike ane dwelling house ane storey high and about thirty six foot long, built with stone and lyme and thatched with straw… ane barn byre & stable with several coalhouses all consisting of twelve bay or thereby built with stone and mud and thatched with straw.” For this, together with 12 acres of marginal land, they paid £416 13 *s* 4*d* Scots plus 20 bolls each of oatmeal and “multured meal,” 24 capons and “half of the land tax and publick burdens.” The same tenants also rented two stone and lime dwelling houses about 60 ft in length, one of which had two stories, with a further three acres of land, in exchange for a further £8 3 *s* 4*d* Scots. James Walker’s possessions included “two milns with a loft in each of them both built with stone and lyme both about seventy foot long and thatched with straw or Beat as also ane maltbarn and kiln with a little loft therein built with stone and mudd and thatched with straw and suchlike a dwelling house consisting of seven bay of length all built with stone and mudd and thatched with straw together with barn byer and stables and severall cotthouses all consisting of about fifty bay or thereby all built with stone and mudd and thatched with straw and only ane storey high.” For this, together with 40 acres of decent land, Walker paid £340 Scots, 20 bolls each of bear (also referred to as bere, is a traditional variety of barley) and oatmeal, three bolls of oats, ten threaves of straw, 39 each of capons and hens and half of the land tax and public burdens.

This information, together with that provided in the account, provides a level of inference in regard to a hierarchy of preferred building materials and methods on the Bannockburn estate. Stone with lime mortar was the walling material of choice for the mills, whilst Dollar and Cowan also maintained three dwellings in the same materials, two of these being fairly commodious. Beyond this, however, all of the outbuildings mentioned, as well as James Walker’s seven bay dwelling and 50 bay cotthouses, were of stone and mud. These tenants clearly held a higher status than the majority of those living on the estate and this was reflected in their maintenance of buildings with stone walls. Mud mortar, however, was deemed more than sufficient for most buildings on their high-investment holdings and it is perhaps telling that James Walker’s cotthouses, unlike most in the account, had walls of stone. The individuals named in the account were above their unnamed lessees in social status, whilst the inclusion of both named and unnamed cottars in the list adds to the suggestion of a graded population that partly operated on a basis of labor services in exchange for parcels of land worked for subsistence (Devine 2006).

## Earth Buildings in the Era of Improvement

The Bannockburn papers provide a uniquely revealing insight into the practice and extent of earth building on an agricultural estate in Central Scotland in the early eighteenth century. Most records that relate directly to earth building in Scotland come from the later eighteenth and nineteenth centuries, however, due to the pertinence of peasant building practices to Improvement ideologies. Attempts to replace the nation’s peasant building stock from the mid-eighteenth century provided a watershed in the gradual loss of most of Scotland’s earth buildings, with political and economic motivations converging over the course of the eighteenth century and reinforced in the following century.

Efforts to deal with the Jacobite rebellions could be seen as one of the catalysts for, and inherently part of, the popularization of later eighteenth century Improvement ideology in Scotland. Comprehensive accounts drawn up by government officials, such as that for Bannockburn, detailed the rental agreements between tenants and forfeited landowners and allowed for an assessment of the population and economy of Scotland, leading to suggested improvements to education, agriculture, industry, and infrastructure. ‘Papers relating to Improvements’ were compiled between 1761 and 1784 and can be aligned with the *Statistical Accounts* and *General Views* in relation to the changes to building practice that they encouraged. Amongst these post-1745 papers are those relating to ‘Leases’ and within them can be found a section entitled “Encouragement for building better houses.” This prescribed in 1761 that “stone, lime and great timbers” should be used in the erection of houses by “masons, wrights and thatchers,” with tenants obliged to undertake repairs “with the like materials” (NAS E730/32 n.d.).

The performance of Scotland’s grain production and trade appears highly erratic in the early eighteenth century. However, unlike the crop failure in 1690, developments in the overall economy of Scottish agriculture appear sufficient to minimize catastrophe with repeated harvest failure in 1739 and 1740 (Smout 1983; Rössner 2011). This period saw the landowning elites pursue a policy of enclosure and adopt increasingly progressive forms of production, allowing labour to be deployed more effectively in activities across many estates. As industrialisation of the wider Scottish economy occurred (Table 1; Fig. 5), further investments in the development of estates were facilitated. The later Improvement era (Table 1) was therefore characterized in part by the acceleration of changes to the agricultural economy. During this period a trend developed amongst Scotland’s political and social elites to perceive earth-built structures in a negative light, with connotations of barbarity, backwardness and poor health. More crucially, activities such as building in earth were regarded as limiting economic growth, given the time that had to be invested in erection and maintenance by tenants themselves and the need for labor to move and adopt different occupations. Therefore, efforts to impose new, ‘better’ buildings with reduced and less seasonally-driven repair and maintenance requirements took hold.

Encouragements to build ‘better’ houses partly manifested in the emergence of planned villages in the Highlands under the auspices of organizations like the British Fisheries Society, which was formed to exploit vast marine resources such as herring and sought to help tame the wild north-west ‘frontier’ through the establishment of Ullapool in 1787 and Tobermory in 1789. These settlements provided a template for new planned towns over the coming decades and were built with stipulations designed to eradicate traditional vernacular building practices and ensure uniformity of standard and regularity in layout. They achieved a high level of success, demonstrating the efficiency with which vernacular traditions could be replaced (Maudlin 2004).

**Fig. 9:**
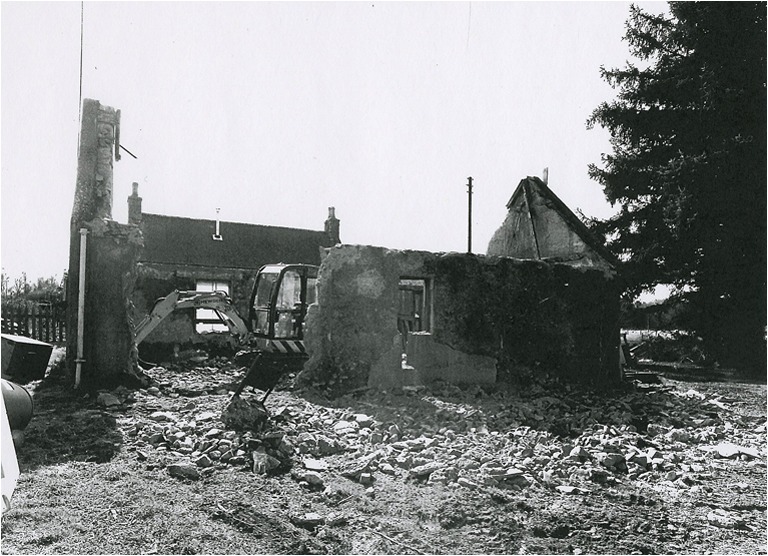
Claywall structure at Upper Haugh, Alford, Aberdeenshire photographed in 2003, with the somewhat amusing comment of the surveyor: “apparently during demolition”. © Crown Copyright: RCAHMS. Licensor www.rcahms.gov.uk

Nevertheless, there remains a great deal of late eighteenth and nineteenth century evidence for the continued existence and erection of mudwall buildings. Indeed, the history of Scottish mudwall building is far longer than would appear if one took the number of remaining examples as reflective of original quantity, a situation demonstrated in Norfolk, England (Longcroft 2006). There are records for the demolition of 59 clay dabbins in Cumbria since the Second World War, before the majority of earth buildings would have already been replaced, and it can be presumed that many more were lost in this later period without documentation (Oxford Archaeology North 2006). In Scotland, the recorded demolition of a mudwall farmhouse at West Freugh, Wigtownshire (Oxford Archaeology North 2005), and the claywall structure at Upper Haugh, Aberdeenshire (Fig. 9) are further evidence of this process.

## Perceptions Post-Improvement

**Fig. 10:**
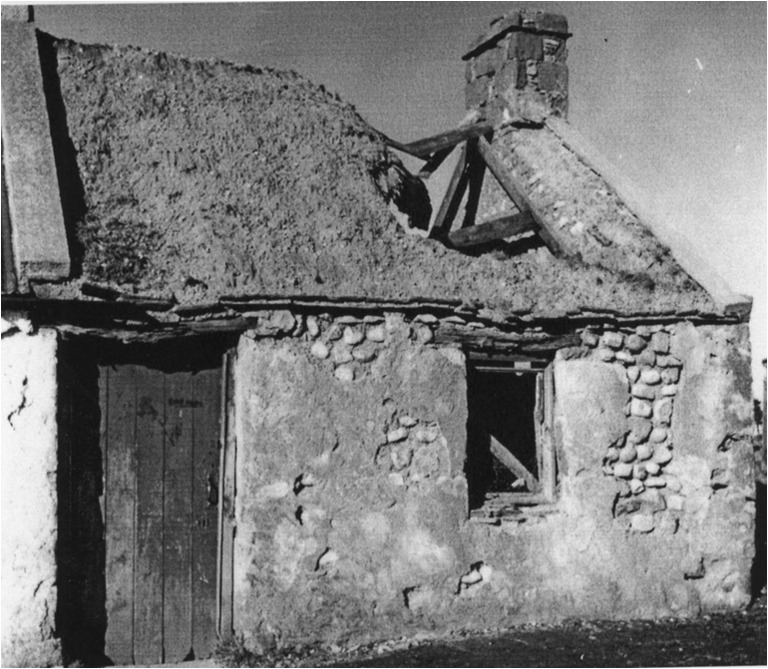
House built in Auchenhalrig Work, Cowfurach, Banffshire (photograph taken by P.J. Nuttgens in 1956, and held by the School of Scottish Studies Archive, University of Edinburgh)

Statements regarding the replacement and improvement of building stock are found repeatedly within the *General Views*, spanning a near 30-year period from the mid-1790s. Nonetheless, sympathetic accounts of traditional methods can be found from within the same body of works. Souter’s *General View* of Banffshire, 1812, follows the typical line in stating that “very considerable improvements have, within these last thirty years, taken place in the erection of farm-houses and offices.” He noted that many farmhouses were “built of stone and clay mortar, having the joining of the stone neatly closed with lime mortar.” And a footnote explains that “In several places in this county the walls of houses and cottages have been built of a composition of mud or clay, mixed with small stones and straw, called *Auchenhalrig Work*. This kind of wall has been recommended for farm-steadings, in situations where stones are not easily procured, and is said to be cheap, substantial, and durable” (Souter 1812). This stands out as a noticeably more pragmatic approach to the assessment of a type of mudwall construction that was idiosyncratic to its locality (Fig. 10). Indeed, Souter provides, in his second appendix, a comprehensive description of the construction process for building a “rood of thirty-six square yards”. The account continues in glowing terms, that when “finished, the Auchenhalrig houses are, out and inside, as ornamental as those built entirely of stone and lime mortar… particularly adapted for dwelling houses, of two stories, and merits attention on account of its cheapness, durability, and warmth, as it excludes every breath of air. The workmanship of a rood costs only about one pound three shillings, and will, when properly built, and well kept under thatch, last for more than a century. Of this there can be no doubt, as in the village of Garmouth, in Morayshire, there are several houses built of these materials, and covered with slate, which have stood upwards of a hundred years, and are at present in excellent condition” (Souter 1812). The account reads as an almost eulogizing assertion and places emphasis on what are presently seen as some of the most redeeming features of mass earth construction.

Earth building continued well into the nineteenth century in Scotland, in spite of the negative influence that Improvement had, physically and metaphorically, on the practice. The combination of vernacular materials in professionally constructed buildings has precedents both documented and in surviving examples, such as a missive letter dating to 1789 relating to the building of a 60 ft long house in Tain with “solid mud, except the corners, door, and window skimshions, Lintols and soles, chimney and chimney heads, Sque and wall tabeling, all of which is to be of the best quarry stones neatly hewed” (MacGill 1909). The combined use of a vernacular material with high quality building stone represents a significant transitional period when the influence of Enlightenment thought was reflected in the built environment. The meeting of traditional and contemporary approaches to construction can also be found in several domestic dwellings in Dumfries and Galloway and in the manorial Kinlochmoidart House, Invernessshire. Such buildings have a stone-faced outward appearance, with internal walls of traditional mudwall, or mud-mortar and boulders (Walker 2009).

The original *Statistical Account of Scotland* was published in 21 volumes between 1791 and 1799 (Sinclair 1791–99), followed by the 52 parts of the *New Account* between 1834 and 1845 (Society for the Benefit of the Sons and Daughters of the Clergy 1845). Both were compiled to describe and quantify the state of the nation using much the same format. They therefore provide insight into the practices and perceptions of mudwall building between the late-eighteenth to mid-nineteenth centuries. An entry for Campbeltown, Argyll, in the *Old* accounts shows the aspirations of Improvement, referring to the abundance of clay and coal as presenting a commercial opportunity through the manufacturing of roof tiles for the Glasgow market that could simultaneously benefit local farmers as they would no longer need to expend straw and labor on thatching, whilst also improving the “cleanliness or comfort” of their dwellings (Sinclair 1791–99). In contrast, some entries demonstrate that mudwall dwellings were not always disregarded and do not demand their replacement. Mudwall dwellings in Elgin, sometimes two stories tall, were deemed comfortable and durable (Sinclair 1791–99), whilst for Dornock, in Dumfriesshire, emphasis was also placed on the quality of living that could be provided by mudwall homes, which were built efficiently through community involvement (Sinclair 1791–99). That the “whole neighborhood” could assemble to help in the building process demonstrates the unspecialized nature of the building method. Whilst it would be best to avoid thinking of such activities as reflecting some kind of collectivist utopia, the social element of vernacular construction is notable. Conversely, however, the report for Peningham, not far from Dornock in the County of Wigton and of comparable geological context, asserts that the population was the “healthiest in the parish” **in spite** of their homes (Sinclair 1791–99). By the time of the *Second Statistical Account*, there is greater uniformity between reports on building quality in rural settings. The continued improvement of the building stock with stone, lime, and slated roofing is lauded, whilst mudwalls receive less favorable reviews than in some of the *Old* accounts. The period between the *First* and *Second Accounts* for St Mungo and Cummertrees north and west of Dornock and Peningham, respectively, saw the replacement of all mud with stone and lime; Dundonald in Ayrshire saw a similar process of replacement of mudwalls with “in many places... elegant architecture;” Dolphinton, in Lanarkshire, saw the removal of a “wretched” landscape in which people dwelt in mudwalled, turf-roofed houses; and in Turriff, Aberdeenshire, farmers’ houses and buildings all became stone and lime with slates, although the cottages remained “of mud, ill-constructed, ill-ventilated, and ill-roofed” with notable exception afforded to those with “well-swept hearth and white-washed wall and sanded floor, [which] give an air of comfort and contentment exceedingly pleasing” (Society for the Benefit of the Sons and Daughters of the Clergy 1845).

The negative perception of vernacular earth-built dwellings prevailed into the twentieth century, although again not universally. Hutcheson’s *Old Stories in Stone and Other Papers*, for example, specifically equated the ‘clay biggins’ of rural Scotland with the intellectual inferiority of their inhabitants: ‘not a going back to barbarism, but an absolute non-advance from barbarism’ (Hutcheson 1927). The lost irony of this assertion is earlier established, however, through reference to one of Scotland’s most intellectually celebrated sons, Robert Burns, and his being born in the same type of ‘clay biggin’ as those rural dwellers disregarded as being absent ‘of all the arts of civilization and literature’ (Hutcheson 1927). Conversely, the celebration of Burns by some of Hutcheson’s contemporaries led to the romanticizing of his humble upbringing and this perhaps influenced later interest in recording and conserving vernacular structures. The romanticizing of the vernacular appears in Sinclair’s *The Thatched houses of the Old Highlands* (1953) and an edited volume, *The Auld Clay Biggin* (Ross 1925), which sought to celebrate the birthplace of the national bard. The collection of poetry and prose in the latter emphasizes the synonymy of Burns’ egalitarian views with his first home, which was typical of the ‘common man.’

## Conclusions

Using both documentary and archaeological evidence the perspectives we present here emphasize the medieval ancestry of Scotland’s surviving eighteenth and nineteenth century mudwall dwellings. We highlight the importance of such sources alongside the surveys cited. Such evidence has previously skewed perceptions of the materials and techniques involved through the implication that non-survival indicates poor-quality and impermanence rather than neglect or removal.

Vernacular mudwall structures were ubiquitous in Scottish landscapes for centuries. The limited archaeological evidence currently available suggests that advances in building practices in the medieval period may have been key to the proliferation of the technique. Further, it was likely better to utilize clay-rich subsoils, which produced homes with favorable thermal qualities, wherever local deposits allowed.

Traditional narratives relating to Scotland’s earth building traditions retain pertinence but we note here that they must also be accompanied by a greater appreciation of earlier perceptions and definitive proof of past proliferation in order to fully appreciate the significance of this all-but-lost craft tradition. The vernacular buildings of the rural majority were negatively perceived for a number of centuries prior to the period of Improvement by outside commentators who starkly contrasted their own civility with the ‘barbarity’ of those who lived in homes constructed of mud. It must be remembered, however, that many commentaries were influenced by politically motivated vested interests that encouraged medieval visitors from England and the continent to emphasize the uncouth nature of Scotland’s society. It is clear that negative perceptions of dwellings were related to more than just the materials used in construction.

That enclaves of historic vernacular mudwall buildings survive in northern Britain despite targeted and sustained efforts at their removal from the Improvement era onwards demonstrates the longevity of these structures of medieval descent. For example, some of the remaining clay dabbins of the Solway Plain have stood for over five centuries despite human encroachment and the notoriously wet climate of western Britain. Whilst mudwall buildings of such date do not remain in Scotland, the still-standing eighteenth and nineteenth century dwellings and agricultural buildings of the Carse of Gowrie north east and south west remain as vestiges of a much older tradition. Although the earlier ubiquity of Scotland’s mudwall buildings has been appreciated for a significant period of time, the early eighteenth century Bannockburn papers provide an invaluable source of evidence to emphasize that this building technique was relied upon to develop the building stock of an estate.

Surviving evidence in the structures built by social elites in medieval Britain reveals a contrasting and pragmatic approach to the use of earthen building materials at the local level, as demonstrated by the presence of mud mortars, earthen partition walls and turf ancillary structures at castle sites, for example. From the mid-eighteenth century, however, Scottish elite society was increasingly concerned with the replacement of vernacular building stock and consequently contributed to the loss of a longstanding tradition justified by notions of the need for modernization, bringing marginal peoples and regions closer to the political center, and in line with the rational thought of the day as a means of encouraging the economic and moral wellbeing of the nation.

## References

[CR1] Brunskill, R. W. (1962). The clay houses of Cumberland. Transactions of the Ancient Monuments Society 10: 57–80.

[CR2] Carruthers, A., and Frew, J. (2003). Small houses and cottages. In Stell, G., Shaw, J., and Storrier S., (eds.), Scotland’s buildings – A compendium of Scottish ethnology Vol. 3, Tuckwell Press, East Linton, pp. 90-107.

[CR3] Devine TM (2006). Clearance and improvement: Land, power and people in Scotland 1700–1900.

[CR4] Dictionary of the Scots Language. (2004). Scottish Language Dictionaries Ltd. http://www.dsl.ac.uk/. Accessed 1 Aug 2016.

[CR5] Dixon, P. (2002). The medieval peasant building in Scotland: the beginning and end of crucks. In Klapste, J. (ed.), The Rural House: from the migration period to the oldest still standing buildings. Ruralia IV: 8–13 September 2001 Bad Bederkesa, Lower Saxony, Germany*,* Brepols, Turnhout.

[CR6] Dyer C (1986). English peasant buildings in the later middle ages (1200-1500). Medieval Archaeology.

[CR7] Fenton A (1968). Alternating stone and turf – An obsolete building practice. Folk. Life.

[CR8] Fenton A (1997). The northern isles: Orkney and Shetland.

[CR9] Fenton A (2008). Country life in Scotland: Our rural past.

[CR10] Fenton A, Walker B (1981). The rural architecture of Scotland.

[CR11] Geddes, G. F. (2013) Vernacular parallels: brochs and blackhouses. Vernacular Architecture 41: 15–27.

[CR12] Guillaud, H., and Houben, H. (2006). Traité de construction en terre, Parenthèses, Marseille.

[CR13] Harrison, J. R. (1989). Some clay dabbins in Cumberland: Their construction and form. Part I. Transactions of the Ancient Monuments Society 33.

[CR14] Harrison, J. R. (1991). Some clay dabbins in Cumberland: Their construction and form. Part II. Transactions of the Ancient Monuments Society 35.

[CR15] Harrison, J. G. (2003). A historical background of Flanders Moss, Scottish natural heritage commissioned report no. 002, Scottish Natural Heritage, Edinburgh.

[CR16] Hume Brown P (1891). Early Travellers in Scotland.

[CR17] Hutcheson A (1927). Old stories in stone and other papers.

[CR18] Jenkins M (2010). Building Scotland.

[CR19] Jennings, N. (2002). The building of the clay dabbins of the Solway plain: Materials and man-hours. Vernacular Architecture 33: 19–27.

[CR20] Klapste, J. (ed.) (2002). The Rural House: from the migration period to the oldest still standing buildings. Ruralia IV: 8–13 September 2001 Bad Bederkesa, Lower Saxony, Germany, Brepols, Turnhout.

[CR21] Longcroft, A. (2006). Medieval clay-walled houses: A case-study from Norfolk. Vernacular Architecture 37: 61–74.

[CR22] MacGill W (1909). Old Ross-shire and Scotland: As seen in the Balnagown documents, the northern counties newspaper and printing and.

[CR23] Maudlin, D. (2004). Regulating the vernacular: The impact of building regulations in the eighteenth-century highland planned village. Vernacular Architecture 35: 40–49.

[CR24] Maxwell I (1996). Building materials of the Scottish farmstead.

[CR25] McGregor, C. (2010). Earth. In Jenkins M. (ed.), Building Scotland*,* John Donald, Edinburgh, pp. 45–55.

[CR26] Murray, H. K., and Murray J. C. (1993). Excavations at Rattray, Aberdeenshire. A Scottish deserted burgh. Medieval Archaeology 37: 109–218.

[CR27] *NAS* E616/1. (n.d.). National Archive of Scotland

[CR28] *NAS* E730/32. (n.d.). National Archive of Scotland

[CR29] *NAS* GD139/110. (n.d.). National Archive of Scotland

[CR30] Noble, R. (1983). Turf-walled houses of the central highlands: An experiment in reconstruction. Folk Life 22: 68–83.

[CR31] Oxford Archaeology North. (2005). MOD west Freugh (formerly RAF west Freugh), Wigtownshire: Archaeological Building Investigation, Oxford Archaeology North, Oxford.

[CR32] Oxford Archaeology North. (2006). Clay buildings on the Cumbria Solway plain, Oxford Archaeology North, Oxford.

[CR33] Parker DE, Legg TP, Folland CK (1992). A new daily Central England temperature series, 1772-1991. International Journal of Climatology.

[CR34] Pennant, T. (1776) A tour in Scotland and voyage to the Hebrides, 1772. B. White, London.

[CR35] Rackwitz, M. (2007). Travels to Terra incognita: The Scottish highlands and Hebrides in early modern travellers’ accounts c.1600-1800*,* Waxmann, Münster.

[CR36] Richards J, Richards M (1994). Timber frame houses in the Scottish countryside.

[CR37] Ross, J. D. (ed.) (1925). The Auld Clay Biggin’: A cluster of prose and poetry celebrating the birthplace of Robert Burns, Standard Print Works, Kilmarnock.

[CR38] Rössner PR (2011). The 1738-41 harvest crisis in Scotland. The Scottish Historical Review.

[CR39] Simmons, A. (ed.) (1998). Burt’s Letters from the north of Scotland as related by Edmund Burt, Birlinn, Edinburgh.

[CR40] Sinclair SJ (1791). The statistical account of Scotland.

[CR41] Sinclair SJ (1795). General view of the agriculture of the northern counties and islands of Scotland; including the counties of Cromarty, Ross, Sutherland and Caithness, and the islands of Orkney and Shetland. With observation on the means of their improvement.

[CR42] Smout TC, Hont I, Ignatieff M (1983). Where had the Scottish economy got to by the third quarter of the eighteenth century?. Wealth and virtue.

[CR43] Smout TC, MacDonald AR, Watson F (2005). A history of the native woodland of Scotland, 1500–1920.

[CR44] Society for the Benefit of the Sons and Daughters of the Clergy (1845). The new statistical account of Scotland by the ministers of the respective parishes, under the superintendence of a committee of the Society for the Benefit of the sons and daughters of the clergy, W.

[CR45] Souter D (1812). General view of the agriculture of the county of Banff; with observations on the means of its improvement. Drawn up for the consideration of the board of agriculture, and internal improvement.

[CR46] Stell, G. (1993). Towards an atlas of Scottish vernacular building. In Cheape H. (ed.), Tools and traditions: Studies in European ethnology presented to Alexander Fenton*,* National Museums of Scotland, Edinburgh, pp. 159–166.

[CR47] Walker B (1977). Clay buildings in north East Scotland.

[CR48] Walker B (1979). The vernacular buildings of north east Scotland: An exploration. Scottish Geographical Journal.

[CR49] Walker B (2006). Getting your hands dirty: a reappraisal of Scottish building materials, construction and conservation techniques. Architectural Heritage.

[CR50] Walker, B. (2009). Clay. In Jenkins M. (ed.), Building Scotland, John Donald, Edinburgh, pp. 57–66.

[CR51] Walker B, McGregor C, Little R (1996). Earth structures and construction in Scotland: A guide to the recognition and conservation of earthen Technology in Scottish Buildings.

[CR52] Walker B, McGregor C, Stark G (2006). Scottish turf construction.

[CR53] Whyte I, Whyte K (1991). The changing Scottish landscape 1500–1800.

[CR54] Wilson MJ, Bain DC, Duthie DML (1984). The soil clays of great Britain: II. Scotland. Clay Minerals.

[CR55] Wrathmell, S. (1984). The vernacular threshold of northern peasant houses. Vernacular Architecture 15: 29–33.

[CR56] Wrathmell, S. (2002). Some general hypotheses on English Medieval peasant house construction from the 7th to the 17th centuries. In Klapste, J, (ed.), The Rural House: from the migration period to the oldest still standing buildings. Ruralia IV: 8–13 September 2001 Bad Bederkesa, Lower Saxony, Germany, Brepols, Turnhout, pp. 175–186.

